# Recent advances in plant disease severity assessment using convolutional neural networks

**DOI:** 10.1038/s41598-023-29230-7

**Published:** 2023-02-09

**Authors:** Tingting Shi, Yongmin Liu, Xinying Zheng, Kui Hu, Hao Huang, Hanlin Liu, Hongxu Huang

**Affiliations:** 1 College of Computer and Information Engineering, Central South University of Forestry and Technology, Changsha, 410004 China; 2 Research Center of Smart Forestry Cloud, Central South University of Forestry and Technology, Changsha, 410004 China; 3 Business School of Hunan Normal University, Changsha, 410081 China

**Keywords:** Computer science, Plant biotechnology

## Abstract

In modern agricultural production, the severity of diseases is an important factor that directly affects the yield and quality of plants. In order to effectively monitor and control the entire production process of plants, not only the type of disease, but also the severity of the disease must be clarified. In recent years, deep learning for plant disease species identification has been widely used. In particular, the application of convolutional neural network (CNN) to plant disease images has made breakthrough progress. However, there are relatively few studies on disease severity assessment. The group first traced the prevailing views of existing disease researchers to provide criteria for grading the severity of plant diseases. Then, depending on the network architecture, this study outlined 16 studies on CNN-based plant disease severity assessment in terms of classical CNN frameworks, improved CNN architectures and CNN-based segmentation networks, and provided a detailed comparative analysis of the advantages and disadvantages of each. Common methods for acquiring datasets and performance evaluation metrics for CNN models were investigated. Finally, this study discussed the major challenges faced by CNN-based plant disease severity assessment methods in practical applications, and provided feasible research ideas and possible solutions to address these challenges.

## Introduction

Plant diseases caused by various organisms that damage plant growth, such as pests, bacteria or fungi, are a major cause of agricultural losses. Reliable and accurate methods for assessing disease severity are essential for effective disease control and minimizing yield loss^1^. There are several ways to assess the severity of plant diseases. The traditional method of determining disease severity is by Visual Assessment, which is highly unreliable due to the similarity of diseases and the diversity of characteristics that are susceptible to external factors and subjective individual differences. Visual Assessment usually needs to be carried out by experienced specialists, which is not efficient, and many farmers do not have access to specialists, making accurate and timely disease severity identification very difficult. In addition, hyperspectral imaging has been used to measure the severity of plant diseases, but this technique requires sophisticated equipment such as sensors and a certain level of expertise, making it costly and inefficient^2^.

In recent years, with the rapid development of computer imaging technology and the continuous improvement of the hardware performance of related electronic devices, computer vision and artificial intelligence (AI) have been widely used in the field of agricultural diagnosis, such as plant species classification, leaf disease identification and plant disease severity estimation^3^. Deep learning has now made significant breakthroughs in the field of computer vision, and CNN has shown excellent performance in plant disease detection applications. Compared to traditional methods, CNN is able to automatically and directly extract features from the input image, eliminating the need for complex image pre-processing and enabling end-to-end detection methods^4^. To date, satisfactory results have been achieved in identifying plant disease species using CNN, but little research has been done in the area of disease severity assessment. This study focuses on the application of CNN for plant disease severity assessment, and systematically reviews the related research to provide reference ideas for further research work.

The remainder of this review is organized as follows: the second part provides an overview of the concepts related to the Visual Assessment of plant disease severity. The third part reviews the history of the development of CNN. The fourth part deals with the specific application of CNN on plant disease severity, illustrating the differences between single-task and multi-task systems. And we focus on the basic working principles of CNN-based plant disease severity assessment methods from three aspects: classical CNN framework, improved CNN architecture, and CNN-based semantic segmentation network, and analyze the advantages and disadvantages of each method. The fifth part summarizes the relevant public datasets and presents CNN performance evaluation metrics. The sixth section discusses the major challenges that CNN-based plant disease severity assessment may face in practical applications, and provides feasible research ideas and possible solutions to these challenges.

## visual assessments

### Definition of plant disease severity

Plant disease severity, defined as the ratio of plant units with visible disease symptoms to the total plant unit (e.g. leaves), is an important quantitative indicator for many diseases^5^. Timely and accurate assessment of disease severity is critical in crop production because disease severity directly affects crop yield and is often used as a predictor to estimate crop loss with excellent accuracy^6^. For example, severity indicators can be used as decision thresholds or disease forecasts to help growers rationalize disease control, such as deciding on the dose and type of pesticide and the time of day to spray.

### Visual assessment methods for plant disease severity

Accurate measurement and evaluation of disease severity is critical to agricultural production. Because it ensures a correct analysis of treatment effects, an accurate understanding of the correlation between yield loss and disease severity, and a reasonable assessment of plant growth stages^7^. Inaccurate or unreliable disease assessment can lead to erroneous conclusions, resulting in the wrong disease management actions, which can further exacerbate losses. Assessment of disease severity is typically done using a variety of scales, including nominal (descriptive) scales, ordinal rating scales, interval (category) scales, and ratio scales^1^. The following is an overview of these scales for visual assessment of disease severity, both qualitative and quantitative.

Qualitative scales.

1. Descriptive scale: This is one of the simplest and most subjective criteria in the disease severity grading scales. The disease is divided into several categories with descriptive terms such as mild, moderate, and severe. Due to the subjectivity and lack of quantitative definitions, the value of this scale is very limited, except for ratings in a specific condition.
2. Qualitative ordinal scale: This is still the descriptive disease scale, but provides more variety in the categories of disease severity levels than the descriptive scale. For example, Xu et al.^8^ assigned a scale of 0–5 to describe the severity of symptoms of zucchini yellow mosaic virus and watermelon mosaic virus to indicate increasing disease severity. This scale is widely used for certain diseases, especially for assessing viral diseases with symptoms that are not easily quantifiable^1, 5^.

Quantitative scales.

1. Quantitative ordinal scale: This scale consists of numbers in known categories, usually the percentage of symptomatic areas. It can be further divided into two types: equal interval and unequal interval. However, equal interval rating scales may give a higher average severity, especially if the actual severity is at the lower end of a category, because the interval is so wide that it is difficult to show differences, leading to an inaccurate rating^9^. Some disease rating scales have unequal intervals. The Horsfall-Barratt scale (H–B scale) is a widely used unequal interval scale. It was developed by Horsfall and Barratt^10^, which effectively alleviates the problem of equal intervals. For example, Bock et al.^11^ used the scale to estimate the severity of citrus ulcer disease. Forbes et al.^12^ used the H-B scale to estimate the severity of potato late blight in the field, etc.
2. Ratio scale: This scale is widely used for visual assessment of severity. The grader measures the percentage of symptomatic organs, defined as 0% to 100%, and rates the severity accordingly. Therefore, the ratio scale places greater demands on the rater to identify and measure the actual disease more accurately.

Although plant disease severity can be assessed by several different methods, both qualitative and quantitative assessment methods tend to result in assessments that are inconsistent with reality due to factors such as the subjectivity of individual raters, the tendency to overestimate disease severity when it is low, and the bias of raters toward 5% whole number intervals^13^. To improve the accuracy of rater estimates, the Standard Area Map (SAD) has long been used as a tool to help estimate plant disease severity^14, 15^. Professional training of raters can also be effective in improving the accuracy of the assessment.

## History of CNN development

**Figure 1:**
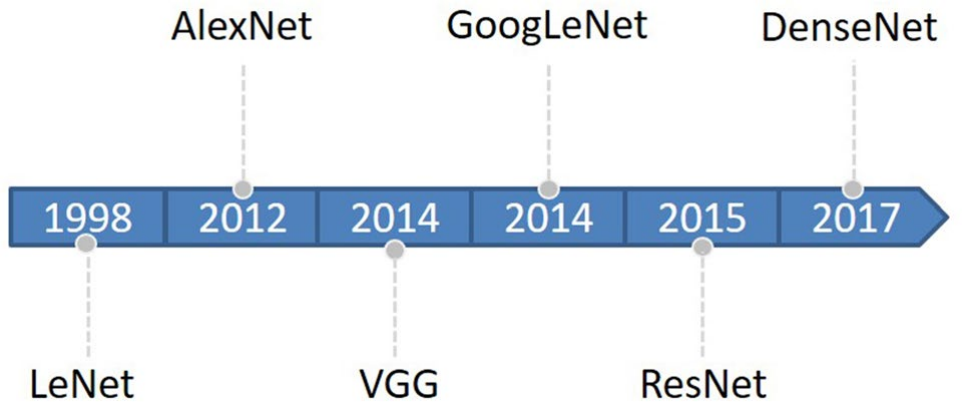
Evolution timeline of CNNs from LeNet to DenseNet.

Deep learning began with the introduction of threshold logic in 1943 and is essentially a process of building computer models that closely resemble human neural networks^16^. CNN is a subset of deep learning that first appeared in the 1980s^17^. In the beginning, the concept of receptive field was developed and later introduced into CNN research^18^. Later, with the introduction of the BackPropagation (BP) algorithm and the training of multi-layer perceptron, researchers tried to automatically extract features instead of manually designing features^19^. LeCun et al.^20^ proposed a CNN architecture called “LeNet-5” using BP networks, which outperformed all other techniques on a standard handwritten digit recognition task at the time. Research on deep neural network models was put on hold due to a number of problems encountered with traditional BP neural networks, such as local optima, overfitting, and gradient disappearance with increasing number of network layers, and the accompanying proposal of some shallow machine models at that time^19^. Until about 2006, Hinton et al.^21^ found that artificial neural networks with multiple hidden layers have excellent feature learning capabilities. Glorot et al.^22^ mitigated the problem of disappearing gradients during training with a normalization method. Attention shifted back to deep learning. In 2012, AlexNet^23^ won the ImageNet Large-Scale Visual Recognition Challenge (ILSVRC), and since then, DL has attracted the attention of more and more researchers, and AlexNet is considered a major breakthrough in the field of deep learning. Next, the CNN architecture continues to evolve, and many algorithms with excellent performance emerge. The main classical CNN networks are LeNet, AlexNet, VGG^24^, GoogLeNet^25^, Resnet^26^, DenseNet^27^, and so on. The evolutionary sequence order from LeNet to DenseNet is shown in Fig. 1.

As CNN evolves, new CNN models are constantly emerging that implement different features. For example, lightweight networks: SqueezeNet^28^, MobileNet^29^, ShuffleNet^30^, Xception^31^, EfficientNet^32^. Target detection networks: R-CNN^33^, Fast R-CNN^34^, Faster R-CNN^35^, YOLO^36^, SSD^37^. Segmentation networks: FCN^38^, SegNet^39^, U-Net^40^, PSPNet^41^, DeepLab^42^, Mask RCNN^43^, etc., they show excellent performance and great research value.

## CNN-based plant disease severity assessment method

CNN has been used with great success to assess the severity of plant diseases. Automatic estimation of plant disease severity based on CNN was first proposed by Wang et al.^44^ in 2017. They used different CNN models to classify apple black rot images with four severity levels and achieved an overall accuracy of 90.4% on the test set, suggesting that CNN is a promising new technique for fully automated plant disease severity classification. Liang et al.^3^ proposed PD^2^SE-Net to implement a multitask system for disease severity estimation, plant species identification, and plant disease classification with overall accuracies of 0.91%, 0.99%, and 0.98%, respectively. Su et al.^45^ combined ResNet-101 network and semantic segmentation to rapidly predict the severity of Fusarium head blight (FHB) in wheat with a prediction accuracy of 77.19%.

### Single-task versus multi-task systems

Deep learning tends to focus on optimizing for specific metrics. In other words, a model or a set of models is often trained to perform the single target task, and such systems are known as single-task systems^46^. On the other hand, there is the concept of multi-task learning (MTL), where multiple tasks can be learned simultaneously if they are linked together^47^. Experimental studies have shown that learning features from multiple related tasks simultaneously is more beneficial than learning them independently in terms of prediction performance. MTL can reduce the risk of overfitting in each task by learning tasks in parallel and thus using more features from different tasks, leading to better generalization of the model^48–50^.

Studies using CNN for plant disease detection include single-task systems that individually identify plant disease species or estimate disease severity. For example, Prabhakar et al.^51^ used ResNet101 to assess the severity of leaf blight in tomato. Zeng et al.^52^ trained six different CNN models to classify the severity of citrus yellow shoot. There are also multitasking systems that perform both tasks simultaneously. For example, José G.M. Esgario et al.^46^ used CNN to implement a classification of coffee leaf disease species and severity grading. Fenu et al.^53^ considered five pre-trained CNN architectures as feature extractors for the classification of three diseases and six severity levels, whose experimental results show that the trained model is robust in automatically extracting disease leaf identification features using a multi-task learning model.

### Application of CNN in plant disease severity assessment

**Figure 2:**
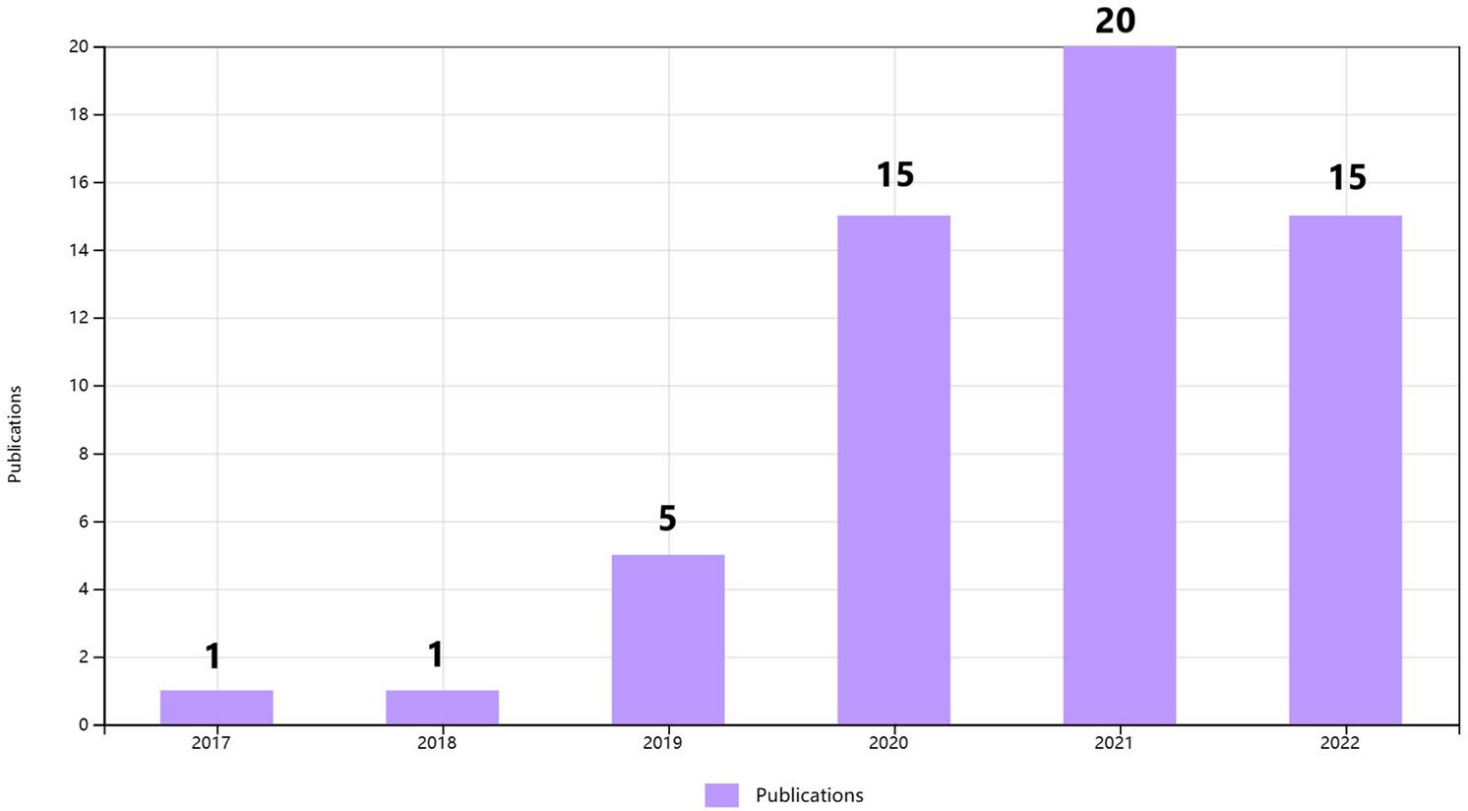
The distribution graph of the publication years of 57 articles based on the keywords of “convolutional neural network” and “plant disease severity” (from Web of Science).

**Figure 3:**
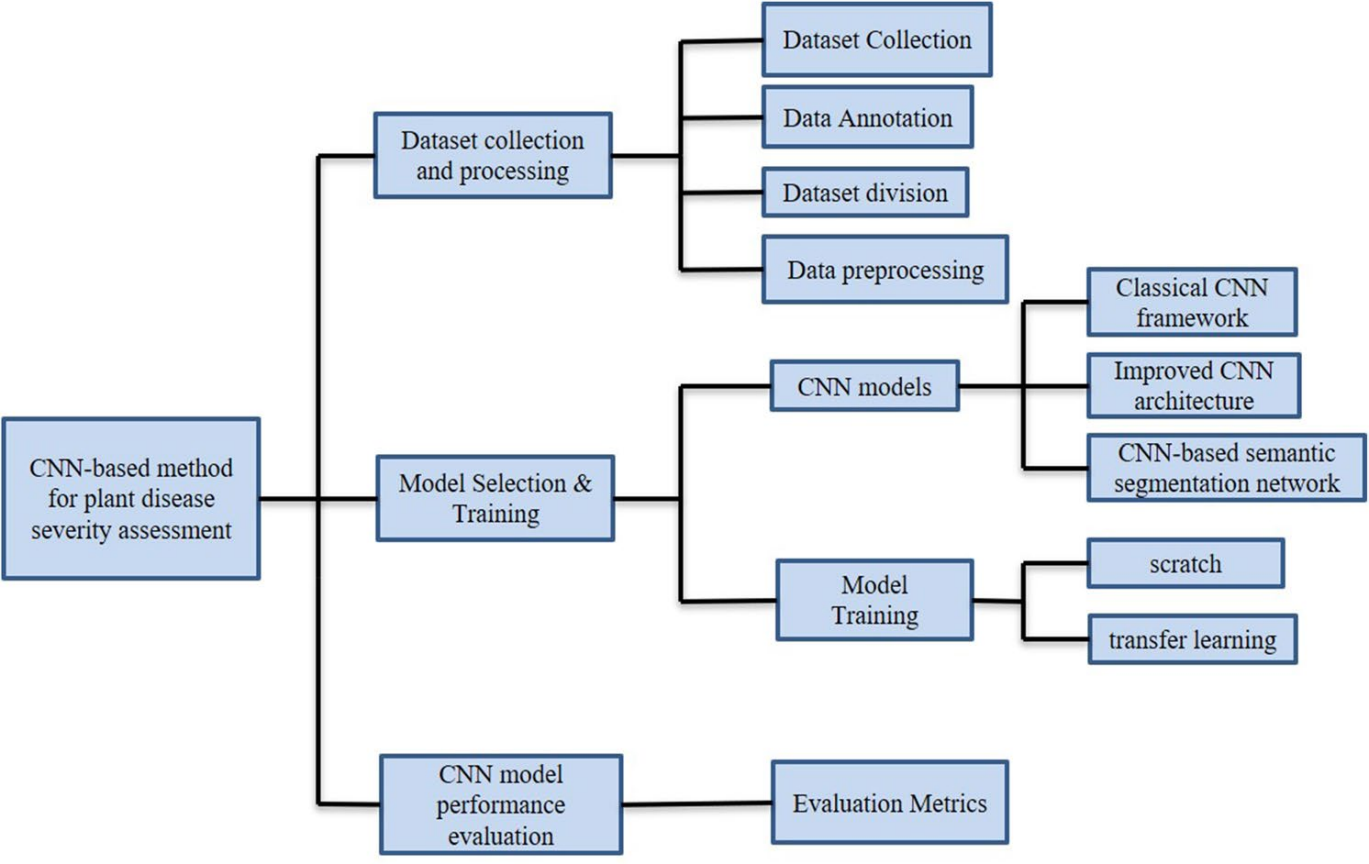
The flowchart of the CNN-based plant disease severity assessment method.

To clarify the specific implementation process of CNN for plant disease severity assessment, 16 high-quality articles that fit the research topic were selected for this study. First, a search was conducted on the Web of Science platform, one of the world's largest and most comprehensive scientific information resources^54^. According to^55^, the process of collecting research sets requires the definition of search terms, so the keywords of “Convolutional neural network” (Topic) and “plant disease severity” (Topic) were entered into Web of Science, and as of 2022, 57 articles were retrieved with the year of publication shown in Fig. 2. Among the 57 papers, 16 papers were selected for specific analysis based on the research object (plant disease) and research method (CNN). On this basis, the most recent research in 2022 is analyzed separately. According to the different CNN network architectures used in these 16 articles, they are further divided into three categories: classical CNN framework, improved CNN architecture, and CNN-based segmentation network. The flowchart of the CNN-based method for plant disease severity assessment method is shown in Fig. 3.

#### Classical CNN framework

10 of the 16 articles are based on the classical CNN framework for implementing severity grading. The 10 studies differ in specific CNN frameworks and research topics, but are similar in that they are CNN-based approaches to assessing plant disease severity. Therefore, they have similarities in the specific implementation process. The process of implementing plant disease severity assessment based on the classical CNN framework can be divided into the following three main steps.

The first step is to collect and process datasets, and is described in four aspects.

1. Dataset characteristics. Of the 10 studies, 6 studies used self-made datasets and 4 studies used the images from PlantVillage images. The self-made datasets can be further divided into two types. One is for images taken under controlled conditions. For example, in^46^, The photos were taken from the abaxial (lower) side of the leaves under partially controlled conditions and placed on a white background. The other is for images taken under natural conditions with a complex background. In contrast, the background of the images in PlantVillage is uniform and homogeneous. Making your own dataset is a time-consuming and expensive process, but it is more in line with what happens in a real environment. A large number of studies have demonstrated that when models trained on controlled images are used to predict images collected from real-world environments, their accuracy is significantly reduced^56–58^. If a public dataset does not meet the needs of a particular study, self-made datasets must be produced.
2. Dataset annotation. One of the necessary conditions for assessing severity is that the records are labeled with different severity levels. Of the 10 articles, 3 were labeled according to the descriptive scale, 1 according to the qualitative ordinal scale, 4 according to the quantitative ordinal scale, 1 article did not indicate the labeling method in the article. For example, in^46^, a quantitative ordinal scale was used. Severity was classified into five levels according to the proportion of diseased leaves: healthy (< 0.1%), very low (0.1–5%), low (5.1–10%), high (10.1%-15%), and very high (> 15%).
3. Dataset division. The dataset is usually divided into three parts: training dataset, validation dataset and test dataset. The training set is used to train the model, the validation set is used to tune the hyperparameters, and the test set is used to evaluate the model performance^59^. The 10 studies all basically used 70% to 85% of the dataset for training. Mohanty et al.^60^ tried five different separation ratios to partition the dataset, and the experimental results showed that using 80% of the dataset for training and 20% for validation was ideal for their data.
4. Data preprocessing. Typically, two preprocessing operations are performed before the images are fed into the CNN. One is to resize the images to match the input layer requirements. For example, The image size in PlantVillage is 256 × 256, and the AlexNet input layer requires a size of 227 × 227, then the original photos need to be resized. This processing is reflected in all 10 studies. Second, the images are normalized to help the model converge faster, significantly improving the efficiency of end-to-end training^61^.

The second step is the model selection and training phase, which is described below in two aspects.

1. CNN framework selection. The CNN frameworks used in the 10 studies include AlexNet, VGG, GoogLeNet, ResNet, DenseNet, MobileNet, Inception, Faster R-CNN, YOLO, EfficientNet, SqueezeNet, Xception, etc. The vast majority of these studies have used multiple CNN frameworks in comparative experiments to determine which model is better at detecting the severity of a particular plant disease under the same training conditions^52^.
2. Training methods. There are two ways to train CNN, one is to start from scratch and the other is transfer learning. Transfer learning refers to adapting a pre-trained network on a large set of images, such as ImageNet (1.2 million images in 1000 classes), to a different task, which is implemented by the underlying CNN learning non-specific features^62^. There are two approaches to transfer learning: feature extraction and fine-tuning. Feature extraction is the process of keeping the weights of a pre-trained model unchanged and then using them to train a new classifier on the target dataset. Fine-tuning involves initializing the model using the weights from a pre-trained model, and then training some or all of the weights on the target dataset^63^. Brahimi et al.^64^ used three approaches of feature extraction, fine-tuning, and training from scratch to train six CNN models. And the results suggested that the fine-tuning models had the highest accuracy and the feature extraction models had the shortest training time. 8 of the 10 studies used transfer learning and only one was trained from scratch. In^44^, the two methods of training models were compared and the results showed that transfer learning alleviated the problem of insufficient training data.

The final step is to evaluate the performance of the CNN models.

The performance of CNN models is obtained by using the test set on the trained model. It is critical that the test set is independent of the training and validation sets, otherwise the evaluation results may be highly biased. Mohanty et al.^60^ trained a model to identify 14 crops and 26 diseases with an overall accuracy of 99.35% on a test set, where there was no clear separation between the validation and test set. When they tested the model on a set of images taken under different conditions than the training images, the model’s accuracy dropped dramatically to 31%. It is worth noting that only 4 of the 10 studies explicitly distinguish three types of datasets. Sibiya et al.^65^ explicitly separated the training set, validation set, and test set. The experimental results showed that the proposed model was neither overfitting nor underfitting, as the model achieved the accuracy of 95.63% on the validation set and a high accuracy of 89% on the test set.

Quantitative assessment of model performance is achieved through evaluation metrics. Evaluation metrics typically include accuracy, precision, recall, mean average precision (mAP), and F1 score based on precision and recall. With the development of deep learning, the performance of CNN models on different datasets has been improved, and various evaluation metrics have been increased. A consistent performance comparison of CNNs from different studies is difficult to achieve because most CNN-based studies for plant disease severity assessment apply to specific datasets, many of which are not yet publicly available and do not provide all the parameters needed to reproduce experiments.

#### Improved CNN architecture

2 of the 16 articles are based on an improved CNN architecture for severity assessment. Comparing the classical CNN framework with the improved CNN architecture, the similarity is that the implementation process is basically the same, and the difference is that the latter uses an improved network based on the classical CNN with the aim of designing a higher performance and more practical system for plant disease diagnosis.

**Figure 4:**
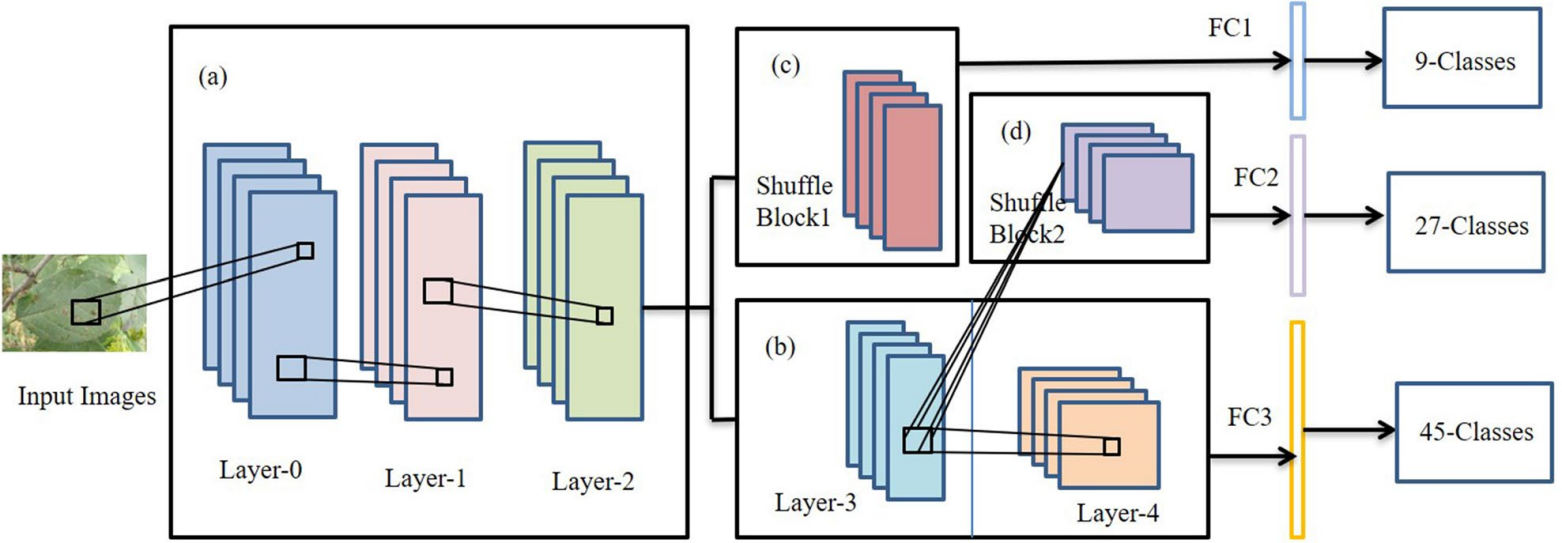
The architecture of PD2SE-Net. Five parts: (**a**) parameter sharing layer; (**b**) the third layer is the parameter sharing layer between the fourth layer and shuffling block 2, and the fourth layer is the high-dimensional feature extractor for severity estimation; (**c**) a feature extractor for plant species recognition; (**d**) feature extractor for plant disease diagnosis; (**e**) fully connected layers^3^.

In^3^, a network, PD^2^SE-Net, was proposed to design a more excellent and practical plant disease diagnosis system. PD^2^SE-Net introduced the ResNet50 network as the base model and integrated the building blocks of ShuffleNet-V2^30^. The PD2SE-Net architecture is shown in Fig. 4. There are two key components of the PD^2^SE-Net that make it so effective. One is the introduction of a residual structure to construct the parameter sharing layers, which allows the model more information to update per batch. Inspired by ShaResNet^66^, ResNet50 was used to build the basic framework and integrated with parameter sharing to reduce the redundant information in the network. The other is the introduction of shuffle units. The ShuffleNet-V2 units were used to extract the feature maps of different plant species and diseases with low computational complexity. Finally, PD^2^SE-Net achieved plant species recognition, disease classification, and severity estimation with overall accuracies of 0.99, 0.98, and 0.91, respectively.

**Figure 5:**
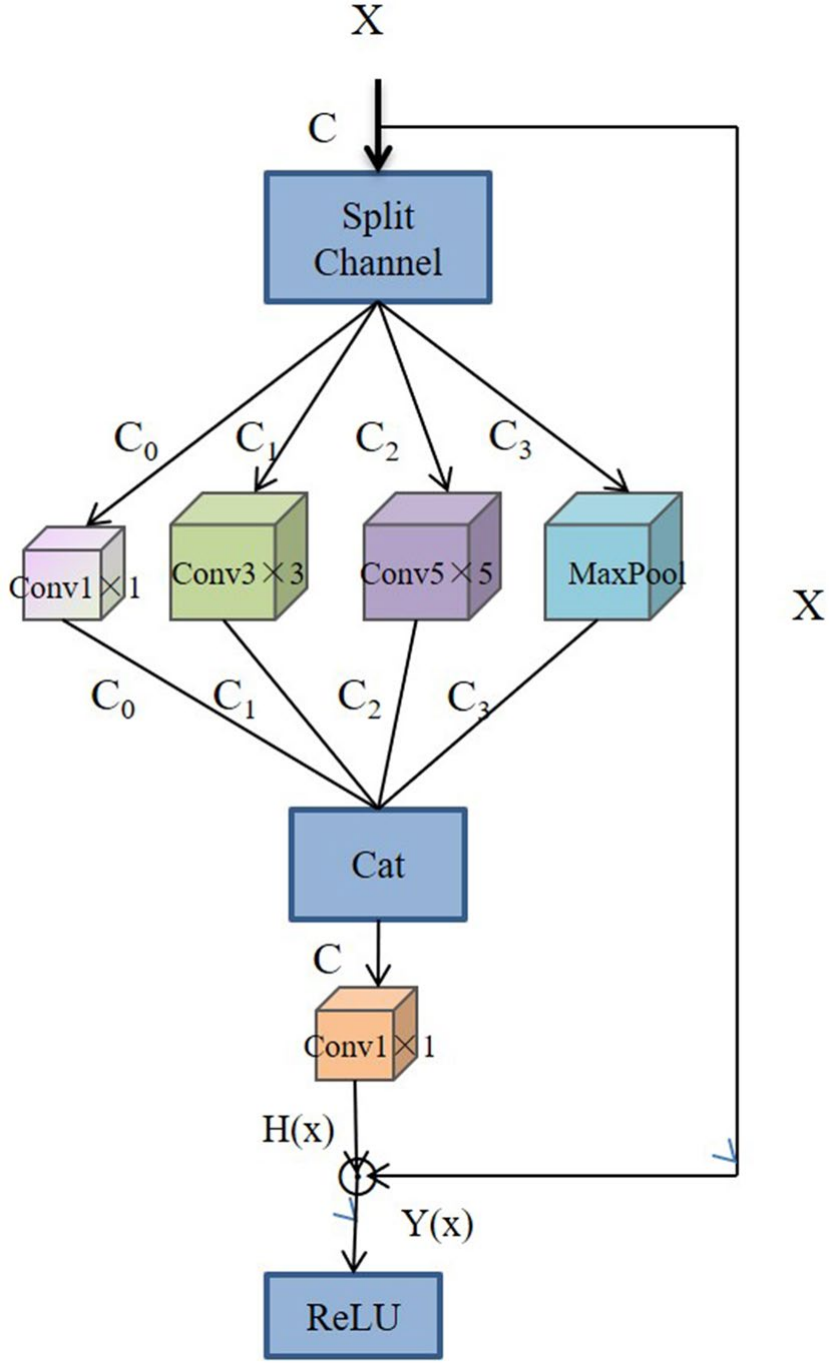
The building block with channel shuffle operation and multi-size convolution module^67^.

Xiang et al.^67^ proposed a lightweight network, L-CSMS, based on residual networks, channel shuffle operation and multi-size module for plant disease severity assessment. Multiple convolution kernels of different sizes were used in the multiscale convolution module to extract different receptive fields in order to obtain robust features and spatial relationships from feature maps^68^. Channel shuffle operation was introduced to enable information communication between different channel groups and to improve accuracy. The channel shuffle operation and the multi-size convolution module were integrated into the building block as a stacked topology, as is shown in Fig. 5. L-CSMS used the residual learning approach of ResNet to build a deep network by stacking modules of the same topology. To validate the performance of the L-CSMS model, Xiang et al.^67^ conducted comparative experiments between the L-CSMS model and ResNet, DenseNet, Inception-V4, PD^2^SE-Net, ShuffleNet, and MobileNet. The results showed that L-CSMS achieved a competitive advantage with fewer parameters, FLOPs, and comparatively good accuracy.

The ways to improve the CNN architecture are different, but the improvements are basically aimed at the same goal, which is to design a more accurate and practical plant disease severity assessment system with better generalization performance. Both studies focused on the residual structure in the ResNet and the channel shuffle.

#### CNN-based semantic segmentation network

Image semantic segmentation has received increasing attention from computer vision and DL researchers, and research work on semantic segmentation using DL techniques continues to evolve. In particular, CNN has far outperformed other methods in terms of accuracy and efficiency^69^. CNN-based segmentation provides not only category information, but also additional information about the spatial location of those categories. The task of semantic segmentation is to label each pixel as a kind of closed objects or a category of regions^70^. CNN-based segmentation theory has been applied to plant disease severity estimation and other related research in agriculture. The main goal of semantic segmentation applied to plant disease severity estimation is to assign appropriate labels to each pixel in order to obtain the percentage of diseased areas required for disease severity estimation.

Typically, the architecture of semantic segmentation is divided into two parts: the encoder network and the decoder network. The encoder is typically based on CNN networks to generate low-resolution image representations or feature maps that are mapped to pixel-level images and then perform prediction and segmentation. The differences between the different semantic segmentation models are often reflected in the decoder networks^70^. The first successful application of deep learning to semantic segmentation was achieved by a fully convolutional network (FCN) constructed by Long et al.^38^. After that, a number of variants of semantic segmentation emerged, such as U-Net, SegNet, DeepLab, and so on.

4 of the 16 articles used the CNN-based semantic segmentation network for plant disease severity assessment. Chen et al.^71^ proposed a BLSNet for estimating the severity of rice bacterial leaf streak (BLS). BLSNet was based on U-Net with the addition of an attention mechanism and multi-scale extraction to improve the accuracy of lesion segmentation. Compared with DeepLabv3+ and U-Net, the experimental results suggested that BLSNet was more suitable for adapting to scale changes of images, and the prediction time of BLSNet was slightly longer than U-Net, but shorter than DeepLabV3+ . Gao et al.^72^ proposed a SegNet-based network to segment potato late blight (PLB) lesions for quantification of the PLB severity. Goncalves et al.^73^ conducted comparative experiments on six semantic segmentation networks (U-net, SegNet, PSPNet, FPN, and 2 variants of DeepLabv3+) applied to three types of plant disease severity estimation (coffee leaf miner, soybean rust, and wheat tan spot).

Although CNNs have provided good results in assessing the severity of plant diseases, the CNN-based semantic segmentation network also has its advantages. The achievement of CNN models for plant disease severity assessment is to directly establish a relationship between severity and samples that is applicable to certain plant diseases, but may not be appropriate for others. For other diseases, the model needs to be retrained. The CNN-based semantic segmentation network is a good solution to this problem by obtaining the percentage of diseased leaf area to reflect the severity through pixel-level segmentation. Previous studies have demonstrated the feasibility of a CNN-based semantic segmentation network for plant disease severity assessment.

#### The research from 2022

In the research of 2022, the methods of using CNN to evaluate the severity of plant diseases can be roughly divided into two categories. One is based on segmentation, and the other is based on improving the CNN, specifically adding the Attention Mechanism. In the segmentation evaluation method, the commonly used segmentation networks include DeepLabV3+ , U-Net, PSPNet and Mask R-CNN. For example, Zhang et al.^74^ used the three-stage method to classify “Huangguan” pears. In the first stage, Mask R-CNN was used to segment “Huangguan” pears from complex backgrounds, and in the second stage, DeepLabV3+ , U-Net and PSPNet were used to segment the “Huangguan” pear spot, and the ratio of the spot area to the pixel area of the “Huangguan” pear was calculated, which was divided into three levels. In the third stage, ResNet-50, VGG-16 and MobileNetV3 were used to obtain the grade of “Huangguan” pear. Liu et al.^75^ also used the idea of stage segmentation. Apple leaves were first segmented from the complex background using the deep learning algorithm, then the disease area of the segmented leaves was identified, and the ratio of the disease area to the leaf area was calculated to evaluate the severity of the disease. Instance segmentation can effectively separate the target from the complex background, which is conducive to dealing with the real environment. In the other method, Attention Mechanism has attracted people's attention. Yin et al.^76^ improved the DCNN based on the addition of multi-scale and attention mechanism, and realized the classification of maize small leaf spot. Liu et al.^77^ introduced a multi-scale convolution kernel and coordinate attention mechanism in SqueezeNext^78^ to estimate disease severity, which was 3.02% higher than the original SqueezeNext model.

## Datasets and evaluation metrics

### Plant disease severity datasets

The correct construction and rational use of plant disease severity datasets is a prerequisite and basis for severity assessment work. Unlike ImageNet, PlantVillage, and COCO in computer vision, there are no large unified datasets for plant disease severity. Plant disease severity datasets can be collected by taking one's own photographs and annotating the images, or by using public datasets and then annotating the images and citing other people's annotated images. With the development and popularity of electronic devices, image collection is typically done through cameras and smartphones. PlantVillage is a common public dataset used in plant disease severity, and common image annotation software is LabelMe, LabelImg, etc. This section provides links to datasets and annotation software used in the 16 studies, as shown in Table 1.

**Table 1:** Datasets and labeling software in the 16 studies, A for Classical CNN framework, B for Improved CNN architecture, C for CNN-based semantic segmentation network, and D for Labeling Software.

	Ref.	Dataset category	Links or references to datasets
A	^44^	Apple leaf black rot	https://plantvillage.psu.edu/ (PlantVillage)
	^52^	Citrus leaf from PlantVillage and crowdAI	https://plantvillage.psu.edu/ https://www.crowdai.org/challenges/1/dataset_files
	^46^	Coffee leaf miner, rust, brown leaf spot (self-made)	https://github.com/esgario/lara2018
	^79^	Cucumber leaves (self-made)	^79^
	^65^	Maize common rust	https://plantvillage.psu.edu/ (PlantVillage)
	^80^	Tea leaf blight (self-made)	^80^
	^51^	Tomato early blight	https://plantvillage.psu.edu/ (PlantVillage)
	^53^	Pear leaf spot, curl, and slug (self-made)	^53^
	^81^	Wheat spike blast (self-made)	https://purr.purdue.edu/publications/3772/1
	^82^	Wheat yellow rust (self-made)	^82^
B	^3^	AI Challenger Global AI Contest	www.challenger.ai, ^83, 84^
	^67^	AI Challenger Global AI Contest	www.challenger.ai, ^3^
C	^73^	Coffee, soybean, and wheat leaves	^73, 85^
	^72^	Potato late blight (self-made)	^72^
	^71^	Rice bacterial leaf (self-made)	^71^
	^45^	Wheat fusarium head blight (self-made)	^45^
D		LabelMe	https://github.com/wkentaro/labelme
		LabelImg	https://github.com/tzutalin/labelImg

### Evaluation metrics

The common evaluation metrics mentioned in the previous model performance evaluation include accuracy, precision, recall, mean average precision (mAP), and F1 score based on precision and recall. Their specific definitions of them are described separately below.

Accuracy, Precision and Recall are expressed by the following equations:

1 $$Accuracy = \frac{TP + TN}{{TP + TN + FP + FN}} \cdot 100\%$$

2 $$Precision = \frac{TP}{{TP + FP}} \cdot 100\%$$

3 $$Recall = \frac{TP}{{TP + FN}} \cdot 100\%$$

In Eqs. (1) and (2), the true positive (TP), with a predicted value of 1 and an actual value of 1, indicates the number of correctly identified lesions. The false positive (FP), with a predicted value of 1 and an actual value of 0, indicates the number of misidentified lesions. The false negative (FN), with a predicted value of 0 and an actual value of 1, indicates the number of lesions not identified. The true negative (TN), with a predicted value of 0 and an actual value of 0, indicates the number of correctly identified non-lesions.

First, it is necessary to calculate the average precision for each category in the data set for mAP.

4 $$P_{average} = \sum\nolimits_{j = 1}^{{N\left( {class} \right)}} {Precision\left( j \right) \cdot Recall\left( j \right) \cdot 100\% }$$

In the equation above, N is the number of all classes and j is the specific class in the dataset.

The average precision for each category is defined as follows:

5 $$mAP = \frac{{P_{average} }}{{N\left( {class} \right)}}$$

The F1 score takes into account both the accuracy and recall of the model, and the equation is:

6 $$F1 = \frac{2Precision \cdot Recall}{{Precision + Recall}} \cdot 100\%$$

## Challenges and future outlook

One of the most important and time-consuming parts of traditional machine learning (ML) methods is the manual feature extraction, while CNN can learn features automatically. Hedjazi MA et al.^86^ addressed the task of visual identification of leaves in images by pre-training a CNN model. The experimental results showed that the pre-trained CNN model outperformed the classical machine learning methods using local binary patterns (LBPs). Bhujel A et al.^87^ designed and tested a semantic segmentation model based on deep learning to detect and measure gray mold on strawberry plants. The results showed that the Unet model outperformed the traditional XGBoost, K-means, and image processing technologies in detecting and quantifying gray mold. Compared with traditional image processing methods and machine learning, plant disease severity assessment has broad application prospects and great development potential, either through the classical CNN framework, improved CNN architecture, or CNN-based semantic segmentation network. Although the technology of plant disease severity assessment is developing rapidly and has gradually moved from academic research to agricultural applications, there is still a certain gap from mature applications in real natural environments, and many problems need to be solved.

### Dataset issues

Dataset problems can be divided into two main aspects: dataset insufficiency and dataset imbalance.

1. Dataset Insufficiency. Adequate datasets are necessary and fundamental for training the network. However, collecting and constructing datasets is an extremely time-consuming, labor-intensive, and costly process. Although there are a number of publicly available datasets for plant diseases, such as PlantVillage, ImageNet, and some publicly available self-made datasets. However, severity annotated datasets are really needed for plant disease severity research. Severity annotation of images is a more tedious process. There are two problems to face in the annotation process, one is the efficiency problem and the other is the accuracy problem. To address the time-consuming and complex annotation process that occurs in manual annotation, a possible solution is to automate the annotation with advanced software, and this automated annotation algorithm is urgently needed. In addition, semi-supervised training and auxiliary labeling methods can be used to increase the speed of agricultural sample processing and help alleviate the workload problem of manual semantic labeling. For the accuracy problem, errors are inevitable whether the annotation is done by manual visual assessment or by software, which is a challenge for future research^1^.
2. Dataset imbalance. The imbalance problem can have a serious impact on the performance of the model, for example, the misclassification rate becomes higher which has been demonstrated in the experiments of many studies^45, 46, 71^. This problem can be well mitigated by data augmentation and weighted loss functions^81^.

### Complex background issues

The dataset can be divided into two types based on image backgrounds: images with uniform backgrounds taken under controlled conditions and images with complex backgrounds taken in natural environments. CNN models are more generalized by using images taken in a natural environment for training compared to a uniform background^56–58^. At the same time, complex backgrounds in images can cause other negative problems. For example, in realistic environments, ground stains resemble disease symptoms, leading to classification errors in the model^72^. Reflections from natural lighting can lead to misclassification of shaded healthy areas or failure to detect disease areas^79^. In addition, it is more common for multiple diseases to occur simultaneously in real-world environments. In^46^, the researchers mentioned that the presence of multiple diseases on a single sheet leaf can significantly change the characteristics of the symptoms, especially when the symptoms overlap, making the system more prone to misclassification. Many studies have shown that when disease symptoms are similar, their error separation rate increases significantly^45, 51, 60^. Due to the problems caused by the complex background, the application of the theoretical results of CNN-based plant disease severity assessment to the actual agricultural production process faces serious obstacles. To solve some of the problems caused by the complex environment, the images can be pre-processed, but this increases the complexity of the whole detection process. For the problem of simultaneous identification and assessment of multiple diseases, researchers in^46^ proposed to alleviate this problem by training a similarity-based architecture that classifies symptoms that are not similar to any disease in the dataset into new classes, such as other classes. This idea has not yet been realized, and further solutions need to be brainstormed.

### Practicality issues

In order to apply theoretical research to practical situations, various solutions have been proposed. As we all know, DCNN is an effective autonomous feature extraction model. Some researches combine deep learning and machine learning methods to build hybrid models. Usually, CNN is used as the feature extraction part and machine learning method is used as the classifier. Saberi Anari et al.^88^ used improved CNN for feature extraction. And multiple Support Vector Machine (SVM) model was used to improve the speed of feature recognition and processing. Kaur et al.^89^ used EfficientNet- B7 for feature extraction. After migration learning, they used logical regression technique to sample the collected features. Finally, the proposed variance technique was used to remove irrelevant features from the feature extraction vector. And the classification algorithm was used to classify the resulting features, and the most discriminative features are identified with the highest constant accuracy of 98.7%. By eliminating irrelevant features, the parameters of the model are greatly reduced. Vasanthan et al.^89^ adopted AlexNet and VGG-19 for feature extraction, and selected the best subset of features by correlation coefficient, and fed them to K-nearest neighbor, SVM, Pulse Neutron Neutron (PNN), Fuzzy Logic, Artificial Neural Network (ANN) and other classifiers. The experimental results showed that the average accuracy of this method was more than 96%.

A server with supercomputing power is needed to ensure that the plant disease severity model built by CNN in the lab is widely used. Cloud computing is essentially a shared pool of computing resources. Cloud computing gathers many computing resources and realizes automatic management through software. Not limited by time and space, anyone who uses the Internet can use the huge computing resources and data centers on the network^90^. PaaS cloud is a concrete implementation of cloud computing. PaaS providers provide many infrastructure and other IT services, and users can access them anywhere through web browsers. The ability to pay for use allows organizations to eliminate the capital expenditures traditionally used for local hardware and software. Lanjewar et al.^91^ deployed the CNN model used to evaluate tea diseases in the PaaS cloud, and the smartphone can access the hyperlink of the deployed model. The image of the tea can be captured by the smartphone camera and uploaded to the cloud. The cloud system automatically predicts the disease and displays it on the mobile display. Lanjewar M G et al.^92^ used the PaaS cloud platform to deploy the CNN model for Curcuma longa detection. While cloud computing brings convenience to us, it inherits the security problems shared by computers and the Internet. In particular, privacy issues, resource theft, attack, and computer viruses. These potential security problems are serious and deserve our attention.

To deploy CNN models on the cloud computing platform, the smaller the size of the model, the better. However, whether the smaller the model can achieve the same evaluation effect is a question worth discussing. Increasing the model size to a certain extent shows better feature extraction effect, such as the comparison between AlexNet and DCNN models such as VGG and GoogLeNet. However, as the model becomes deeper and larger, the degradation problem occurs. The residual structure of ResNet effectively mitigates this problem. More and more lightweight networks have been proposed. Their efficiency may not be the best, but it is worth trading a small amount of effectiveness for a large amount of efficiency. Liu et al.^77^ improved SquezeNext and performed comparative experiments with ReseNet-50, Xception, and MobileNet-V2. The experimental results showed that the accuracy of the proposed method was slightly better than that of Xconcept, while the model size was only 2.83 MB, which was only 3.45% of Xconcept. Model structure is a key factor to balance model size and performance.

Although CNN has shown excellent performance and great potential in assessing the severity of plant diseases, CNN also has its own limitations, such as translation invariance, pooling layer leading to information loss, and inability to obtain global features well. As a possible contribution and future work, new techniques that have become quite popular recently, such as vision transformers. The main feature of vision transformers^93, 94^ is the self-attention mechanism, which can capture the global information well. As far as I know, no research has applied it to severity estimation.

At present, some problems still have not found appropriate solutions, which indicates that the current research on automatic assessment of plant disease severity is far from mature and perfect practical application, which requires more scholars to continue to struggle to study the unsolved problems. The review article of our group on “Recent Advances in Plant Disease Severity Assessment Using Convolutional Neural Networks” provides some references for related types of research work. And more importantly, it can provide new ideas for the subsequent research work.

## Data Availability

All data generated or analysed during this study are included in this published article and its supplementary information files.
